# Supplementary material for: Complete genetic and epigenetic architecture of D4Z4 macrosatellites in FSHD, BAMS, and reference cohorts with D4Z4End2End
Source: Genome Res. 2026 Apr;36(4):827–48. doi: 10.1101/gr.280907.125 (PMC13138017; doi:10.1101/gr.280907.125)
Supplement: Supplement 1 [file Supplemental_Material.pdf]

	Patient samples (# of individuals)	Targeted/ WGS approach	Alleles studied			Methylation	Mosaicism, complex alleles (# of alleles)	FSHD2 genes
			Hap.	Range of array size (# of alleles)	Max. # of spanning reads for a single allele			
Hiramuki et al. 2022	FSHD1 (12)<br>FSHD2 (2)	Cas9-targeted<br>- p13E-11, A-sequence	4qA<br>10qA	1-13 RU (15)<br>7-13 RU (9)	15 (2RU)<br>6 (8RU)	Per-RU for 4qA and 10qA reads; promoter + gene body for distal RU	N/A	N/A
Yeetong et al. 2023	FSHD1 (7)<br>Unaffected parents (6)<br>Controls (10)	WGS	4qA<br>10qA	1-5 RU (7)<br>6-9 RU (3)	6 (2RU)<br>3 (6RU)	N/A	N/A	N/A
Butterfield et al. 2023	FSHD1 (8)<br>FSHD2 (2)<br>*Controls (1)**	Cas9-targeted<br>- p13E-11, pLAM<br>- D4Z4<br><br>*Publicly-available WGS**	4qA<br><br>10qA<br><br>*4qB**	2-25 RU (11)<br>*42 RU (1)**<br>7-24 RU (12)<br>*20-21 RU (2)**<br>*16 RU (1)**	78 (5 RU)<br><br>63 (7 RU)	Base-level and per-RU for 4qA, 10qA and 4qB alleles	N/A	N/A
Li et al. 2024	FSHD1 (1)	WGS	4qA	5 RU (1)	3 (5 RU)	N/A	N/A	N/A
Lemmers et al. 2024	FSHD1 (2)<br>Unaffected mother (1)	Cas9-targeted<br>- p13E-11, pLAM	4qA	17+2 RU (1)<br>17+9 RU (1)<br>2+10 RU (1)	Spanning reads obtained for individual arrays and spacer, but none spanning both arrays	Base-level and per-RU for 4qA *in-cis* duplication alleles	Duplication (3)	N/A
Huang et al. 2024	FSHD1 (12)<br>Controls (11)<br>iPSCs (6) from FSHD1 and controls	WGS	4qA<br>4qB<br>10qA	2-21 RU (23)<br>7-20 RU (7)<br>6-23 (21)	8 (4RU)<br>4 (7RU)<br>10 (6RU)	Base-level and average for distal RU for 4qA alleles	Mosaicism (1)	*SMCHD1*, *DNMT3B*, *LRIF1*
Wang et al. 2024	FSHD1 (1) – prenatal diagnosis via amniocentesis	WGS	4qA<br>4qB	4 (1)<br>8 (1)	2 (4RU)<br>2 (8RU)	Per-RU for 4qA and 4qB alleles	N/A	N/A
Our study	FSHD1 (4)<br>FSHD2 (4)<br>BAMS (2)<br>Controls (2)	Cas9-targeted<br>- 4q and 10q<br>- FSHD2 gene panel<br>WGS	4qA<br>4qB<br>10qA<br>10qB	1-42 RU (14)<br>13-26 RU (5)<br>6-36 (20)<br>13-19 (3)	116 (2RU)<br>79 (13RU)<br>163 (7RU)<br>3 (17RU)	Base-level and per-RU for 4qA, 4qB, 10qA, 10qB alleles (incl. duplicated arrays and upstream inverted RUs)	Mosaicism (2)<br>Duplication (2)<br>Triplication (1)<br>Upstream inverted array (1)	*SMCHD1*, *DNMT3B*, *LRIF1*

Supplementary Table S1. Comparison of our study with previous Nanopore-based studies for FSHD. Our study extends upon previous studies by analysing samples from BAMS patients, including guide RNAs for FSHD2-associated genes for Cas9-targeted sequencing, capturing longer full-length D4Z4 alleles with higher coverage, and analysing the structure and methylation of full-length complex alleles, including those with duplications and triplications of the D4Z4 array.

	Haplotypes from Nanopore raw spanning reads (full D4Z4 units)	HPRC assembly				
		Scaffold	p13E11	pLAM/ B-sequence	No. of full D4Z4 units Start / middle / end / full array	
**HG00621**	4qAS 13 RU<br>4qB 15 RU<br>10qA 10 RU<br>10qA 19 RU	HG00621#2#JAHBCC010000040.1	Y	--	8	start
		HG00621#2#JAHBCC010000132.1	Y	pLAM	19	full
		HG00621#2#JAHBCC010000194.1	--	B-sequence	12	end
		HG00621#1#JAHBCD010000073.1	Y	pLAM	13	full
		HG00621#1#JAHBCD010000149.1	Y	pLAM	10	full
**HG00735**	4qAS 34 RU<br>4qB 46 RU<br>10qA 4 RU<br>10qA 31 RU	HG00735#2#JAHBCG010000093.1	Y	--	11	start
		HG00735#2#JAHBCG010000109.1	Y	pLAM	4	full
		HG00735#2#JAHBCG010000195.1	--	B-sequence	18	end
		HG00735#2#JAHBCG010000230.1	--	--	15	middle
		HG00735#1#JAHBCH010000030.1	Y	--	6	start
		HG00735#1#JAHBCH010000093.1	Y	--	14	start
		HG00735#1#JAHBCH010000168.1	--	pLAM	5	end
		HG00735#1#JAHBCH010000258.1	--	pLAM	7	end
**HG00741**	4qB 10 RU<br>4qAS 38 RU<br>10qA 20 RU	HG00741#2#JAHALX010000140.1	Y	B-sequence	10	full
		HG00741#2#JAHALX010000136.1	Y	--	11	start
		HG00741#2#JAHALX010000243.1	--	pLAM	6	end
		HG00741#1#JAHALY010000013.1	Y	--	10	start
		HG00741#1#JAHALY010000157.1	--	pLAM	5	end
		HG00741#1#JAHALY010000230.1	Y	--	4	start
		HG00741#1#JAHALY010000244.1	--	pLAM	8	end
**HG01106**	4qB 15 RU<br>4qA 28 RU<br>10qA 17 RU	HG01106#2#JAHAMB010000127.1	Y	--	6	start
		HG01106#2#JAHAMB010000188.1	--	B-sequence	9	end
		HG01106#2#JAHAMB010000214.1	--	pLAM	5	end
		HG01106#1#JAHAMC010000060.1	Y	--	7	start
		HG01106#1#JAHAMC010000067.1	Y	--	7	start
		HG01106#1#JAHAMC010000178.1	--	pLAM	18	end
		HG01106#1#JAHAMC010000192.1	--	pLAM	5	end
**HG01175**	4qB 33 RU<br>4qAS 52 RU<br>10qA 8 RU<br>10qB 20 RU	HG01175#2#JAHALZ010000099.1	Y	pLAM	3	full
		HG01175#2#JAHALZ010000122.1	Y	--	3	start
		HG01175#2#JAHALZ010000265.1	--	pLAM	8	end
		HG01175#1#JAHAMA010000091.1	Y	--	6	start
		HG01175#1#JAHAMA010000128.1	Y	B-sequence	9	full
		HG01175#1#JAHAMA010000263.1	--	B-sequence	20	end

Supplementary Table S2. Comparison of D4Z4 genotypes determined using raw Nanopore sequencing data to draft assemblies from the Human Pangenome Reference Consortium (HPRC). The haplotyping pipeline was used to process publicly-available raw Nanopore sequencing data generated by the HPRC (Liao et al. 2023) for 5 lymphoblastoid cell lines (LCLs) from the 1000 Genomes Project. 4q and 10q haplotypes were able to be determined from spanning reads present in the raw data. D4Z4 arrays within the corresponding HPRC draft assemblies were then assessed for their number of repeat units, and for whether they spanned the full D4Z4 array.

SAMPLE	Sex	D4Z4 haplotypes	*SMCHD1* genotype	%5mC DR1
**Control**				
**AG09309**	F	>10	No mutation	Not known
**AG10803**	M	>10	No mutation	Not known
**FSHD1**				
**12566** [a,b]	F	4qA 3RU	No mutation	36.8%
**FSHD1_3 (TalF** [a,b]**, 37-I$_1$** [c]**)**	M	4qA 2RU<br>4qB 18RU<br>10qA 36RU<br>10qA 15+2+6RU (cis-triplication) [c]	No mutation	45.8%
**17706** [a,b]	F	4qA 2RU (25%)<br>4qA 15RU<br>4qA 37RU<br>10qA 6RU<br>10qA 14RU	No mutation	38.6%
**19187**	F	4qA 2RU (50%)<br>4qA 22RU<br>4qA 32RU<br>10qA 14RU<br>10qA-cis duplication	No mutation	45.7%
**FSHD2**				
**11440** [d]	M	4qA 11RU<br>4qA 34RU<br>10qA 15RU<br>10qA 17RU	c.2338+4A>G	16.4%
**15166** [d]	F	4qA 11RU<br>4qB 21RU<br>10qB 13RU<br>10qB 20RU	c.4608_4614dup<br>p.Ala1539Tyrfs*6	11.3%
**34140**	F	4qA 29RU<br>4qA 30RU<br>10qA 17RU<br>10qA 19RU	p.L1031	37%
**11491** [d]	F	4qA 22RU<br>4qA 22RU<br>10qA 6RU<br>10qA 6RU	c.5476+3A>G	9.8%
**BAMS**				
**BAMS1** [e]	M	Not known	c.407A>G<br>p.E136G	Not known
**BAMS9** [e]	M	Not known	c.1259A>T<br>p.D420V	Not known

Previously reported in: a. Dion et al. 2019; b. Gaillard et al. 2019; c. Delourme et al. 2023; d. Gérard et al. 2024; e. Gordon et al. 2017

Supplementary Table S3. Previous genotyping and methylation data for control, FSHD and BAMS fibroblasts. FSHD and BAMS patients were diagnosed based on clinical findings and clinical genetic testing. D4Z4 genotyping was performed via Southern Blot and/or molecular combing. Methylation analysis was performed via bisulfite sequencing using PCR primers specific for the DR1 region.

D4Z4_up_1	GATACCGACAGCAATAGTCC
D4Z4_up_2	GTTGTGAAGTTAGAAGGTGC
D4Z4_A_down_1	GTATGCTGCGGGTTGTGGGG
D4Z4_A_down_2	GAACACACTACCTTTCCATG
D4Z4_B_down_1	GGAATGTATAATACTTCTGC
D4Z4_B_down_2	GAGTCTACAGTAGTGTTCGA
smchd1_p1_up	CCAACTTCGCGAGGGCCGAG
smchd1_p1_down	AGGAGATCGAACATGACAAC
smchd1_p2_up	CTAAGCACCCTACTTTAATT
smchd1_p2_down	GAGCAGTGCAAGAGTGAAAG
smchd1_p3_up	CATTGACCCATACTTCGAGA
smchd1_p3_down	TTAGGAGATATCATTTACGA
smchd1_p4_up_1	TTACCTTCTTAAGCAGTGCA
smchd1_p4_up_2	GTGATTTCATCCTTTGACCA
smchd1_p4_up_3	ACACTATTTGTACTCTCTTG
smchd1_p4_down	AATGATAACCCACTGCCATA
dnmt3b_exon1_up	CCTGTCCTTAGTTTACTGCG
dnmt3b_exon1_down	GCAATGAACTTGGCGAGACA
dnmt3b_rest_up	GTGAAAATCCCGCTTCAGGC
dnmt3b_rest_down	GGGCTTGCCCGTCTGTCTTA
lrif1_up	ACTCACTTAAAAGCTCTACA
lrif1_down	GACTAATTAATAGTGCTGCG

Supplementary Table S4. Guide RNAs (gRNAs) used for Cas9-targeted sequencing. gRNAs were designed using CHOPCHOP v3 (Labun et al. 2019) (GRCh38 or CHM13 T2T v1.1, CRISPR/Cas9, nanopore enrichment, 'Doench et al. 2014 – only for NGG PAM').

4AS-F	CCCGCCCGGGCCCCTGCA
4AL-F	CGAGGACGGCGACGGAGAC
4AM-F_345	AAAGCGGTCCTCGGCCTC
4AM-F_565	ACACCGGGGACGCTGAG
PAS-R	GATCCACAGGGAGGGGGCATTTTA

Supplementary Table S5. PCR primers used for 4qAS/4qAM/4qAL genotyping. 4AS-F, 4AL-F and PAS-R are from Lemmers et al. 2018. 4AM-F_345 and 4AM-F_565 were designed using Primer-BLAST (Ye et al. 2012) based on the consensus sequence for the 19187 4qAM allele, and produce 345bp and 565bp amplicons when used with PAS-R, respectively.

Cell line	4q		10q	
	Hap.	RUs	Hap.	RUs
**HG00099**	4qB	23	10qA	7
	4qB	2.5* + 35	--	--
**HG00105**	4qAS	37	10qA	22
	--	--	10qA	27
**HG00110**	4qB	23	10qA	16
	4qAL	30	--	--
**HG00121**	4qAL	18	10qA	14
	--	--	--	--
**HG00127**	4qB	19	10qA	11
	--	--	10qA	31
**HG00136**	4qB	17	10qA	15
	--	--	10qA	30
**HG00151**	4qAS	31	10qA	12
	--	--	10qA	31
**HG00410**	4qB	12	10qA	21
	4qAS	29	--	--
**HG00525**	--	--	10qA	34
	--	--	10qA	41
**HG00631**	4qAS	13	10qA	7
	--	--	10qA	19
**HG00675**	4qAS	21	10qA	10
	4qB	≥18 + 3^	10qA	13
**HG00706**	4qB	24	10qA	7
	--	--	--	--
**HG00728**	4qB	13	10qA	10
	--	--	10qA	14
**HG01046**	4qB	22	10qA	25
	4qAS	32	--	--
**HG01122**	4qB	40	10qA	23
	--	--	10qA	9
**HG01281**	4qB	30	10qA	37
	4qAL	26	--	--
**HG01369**	4qB	23	10qA	8
	4qB	20	--	--
**HG01372**	4qAS	21	10qA	12
	4qAS	31	10qA	17
**HG01395**	--	--	10qA	18
	--	--	--	--
**HG01414**	4qB	26	10qA	9
	--	--	10qA	11
**HG01468**	4qAS	27	10qA	11
	--	--	--	--
**HG01615**	--	--	10qA	22
	--	--	10qA	15
**HG01695**	4qB	20	10qA	6
	4qAS	26	10qB	19
**HG01790**	4qB	23	10qA	38
	4qAS	43	--	--
**HG01797**	4qB	14	10qA	7
	4qB	28	10qA	25
**HG01801**	--	--	10qA	11
	--	--	10qA	23
**HG01812**	--	--	10qA	13
	4qAS	26	10qA	17
**HG02185**	4qAS	26	10qA	21
	4qB	12	10qA	24
**HG02252**	4qAS	29	10qA	10
	--	--	--	--
**HG02282**	--	--	10qA	8
	--	--	--	--

*Upstream inverted D4Z4 array
^Downstream non-inverted D4Z4 array

Supplementary Table S6. D4Z4 haplotypes for 30 B-lymphoblastoid cell lines (B-LCLs) from the 1000 Genomes Project. Haplotypes were determined from spanning D4Z4 reads within publicly-available raw Nanopore sequencing data from the 1000 Genomes Project ONT Sequencing Consortium (1KGP-ONT) (Gustafson et al. 2024), as identified by D4Z4End2End. Blank cells indicate that no spanning reads were identified for that allele.

SAMPLE	Cas9	WGS
**Control**		
AG09309		R10.4.1, SQK-ULK114
AG10803		R10.4.1, SQK-ULK114
**FSHD1**		
12566	R9.4.1, SQK-CS9109	R9.4.1, SQK-ULK001
FSHD1_3		R9.4.1, SQK-ULK001
17706	R9.4.1, SQK-CS9109	R9.4.1, SQK-ULK001
19187	R10.4.1, SQK-ULK114*<br>R9.4.1, SQK-CS9109*	
**FSHD2**		
15166		R10.4.1, SQK-ULK114
34140		R10.4.1, SQK-ULK114
11491		R10.4.1, SQK-ULK114
11440	R9.4.1, SQK-CS9109	
**BAMS**		
BAMS-1		R9.4.1, SQK-ULK001^<br>R10.4.1, SQK-ULK114^
BAMS-9	R9.4.1, SQK-CS9109	R9.4.1, SQK-ULK001

*Initial sequencing for 19187 using R10.4.1 with SQK-ULK114 showed poor efficiency and read quality, therefore additional sequencing was performed using R9.4.1 with SQK-CS9109

^An initial sequencing run for BAMS-1 on R9.4.1 with SQK-ULK001 yielded low coverage due to a low DNA input amount, therefore an additional run was performed using R10.4.1 and SQK-ULK114

Supplementary Table S7. ONT flow cell and kit versions used for Cas9-targeted and whole-genome sequencing (WGS) of control, FSHD and BAMS fibroblasts. Libraries were prepared using SQK-ULK001 or SQK-CS9109 for loading onto R9.4.1 PromethION flow cells (FLO-PRO002), or SQK-ULK114 for loading onto R10.4.1 flow cells (FLO-PRO114M), and sequenced on a PromethION P24.

SMCHD1

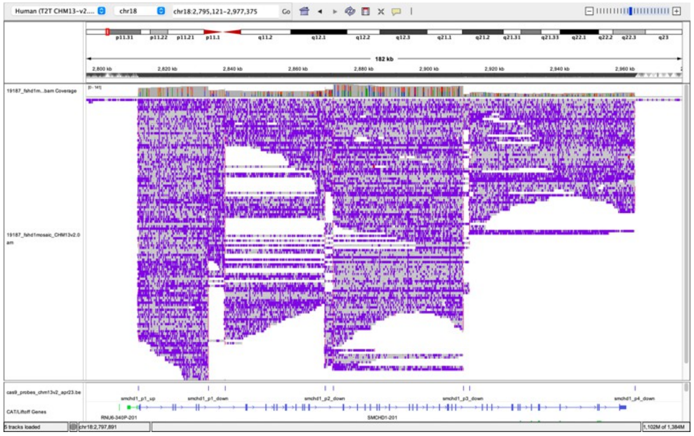

DNMT3B

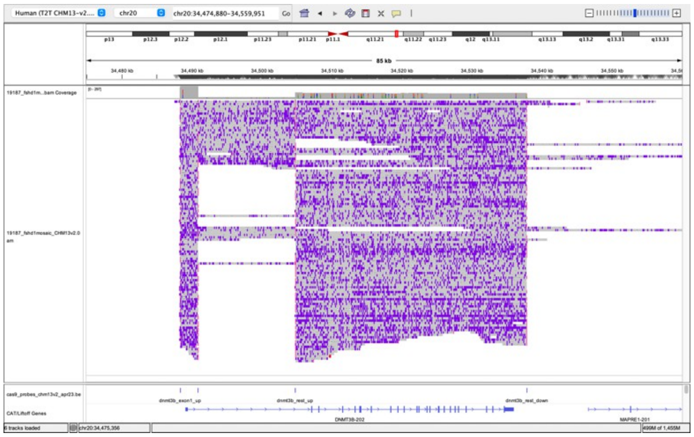

LRIF1

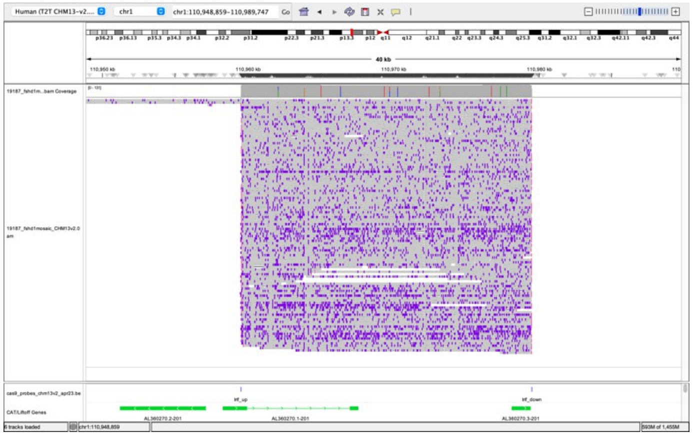

Supplementary Figure S1. Coverage of FSHD2 gene panel using Cas9-targeted sequencing. IGV screenshots of *SMCHD1*, *DNMT3B* and *LRIF1* coverage from Cas9-targeted sequencing of sample 19187 fibroblasts. Locations of Cas9 gRNAs (Supplementary Table S4) are shown below the alignments.

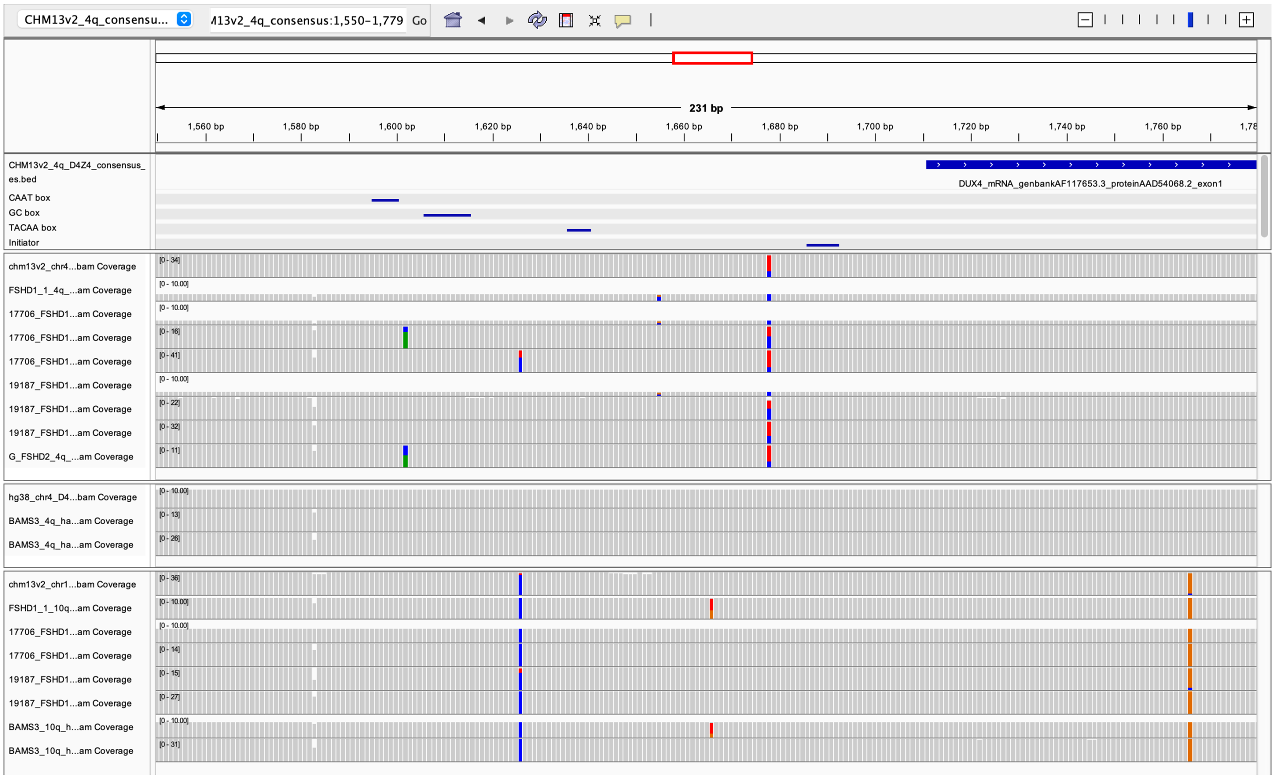

Supplementary Figure S2. Sequence variants in the *DUX4* promoter region for 4qA, 4qB and 10qA alleles. The locations of known *DUX4* promoter elements (Dixit et al. 2007), are shown in the top panel, alongside the start of *DUX4* exon 1. Coverage tracks for alignments of D4Z4 units extracted from full-length D4Z4 consensus sequences are shown for 4qA alleles (second panel), 4qB alleles (third panel) and 10qA alleles (bottom panel), displaying 4qA-, 4qB- and 10qA-specific SNVs.

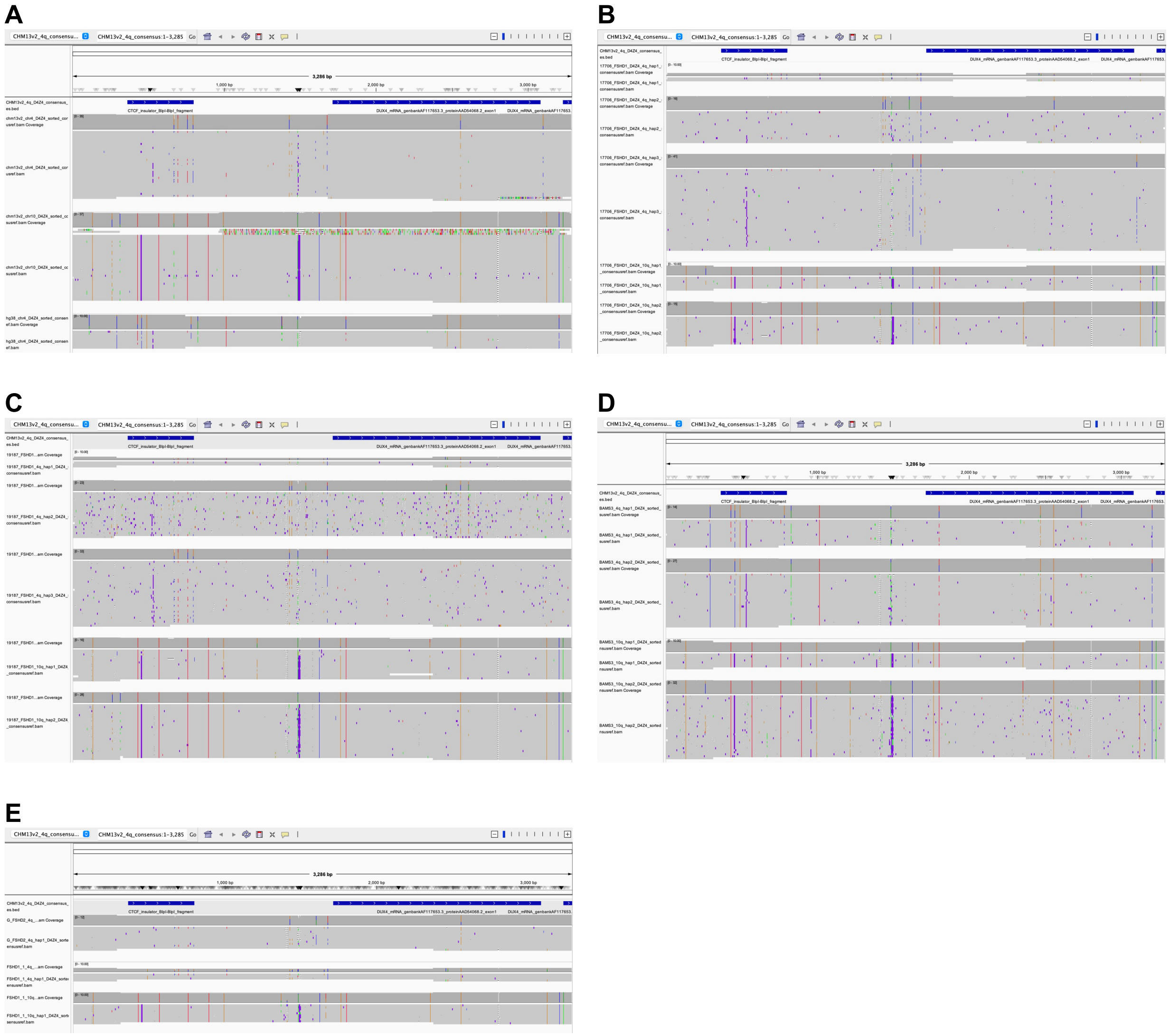

Supplementary Figure S3. Analysis of inter-D4Z4 genetic variation for 4qA, 4qB and 10qA alleles. Each panel shows alignments of individual D4Z4 units from the consensus sequences of full-length D4Z4 alleles, against a 4qA-type reference sequence (derived from CHM13v2.0, see Methods). Within each alignment track, full-length D4Z4 units are arranged from top to bottom in order of proximal to distal position within the array. D4Z4 units from (A) the 4qA and 10q arrays from CHM13v2.0, and the 4qB array from GRCh38 (B) sample 17706 (FSHD1, mosaic sample) (C) sample 19187 (FSHD1, mosaic sample); note that the 10qA 15RU allele ('10q_hap1') contains a truncated internal D4Z4 unit, and the proximal partial D4Z4 unit is shown in the same row as this truncated repeat (D) sample BAMS9 (E) samples 11440 (labelled as 'G_FSHD2') and 12566 (labelled as 'FSHD1_1').

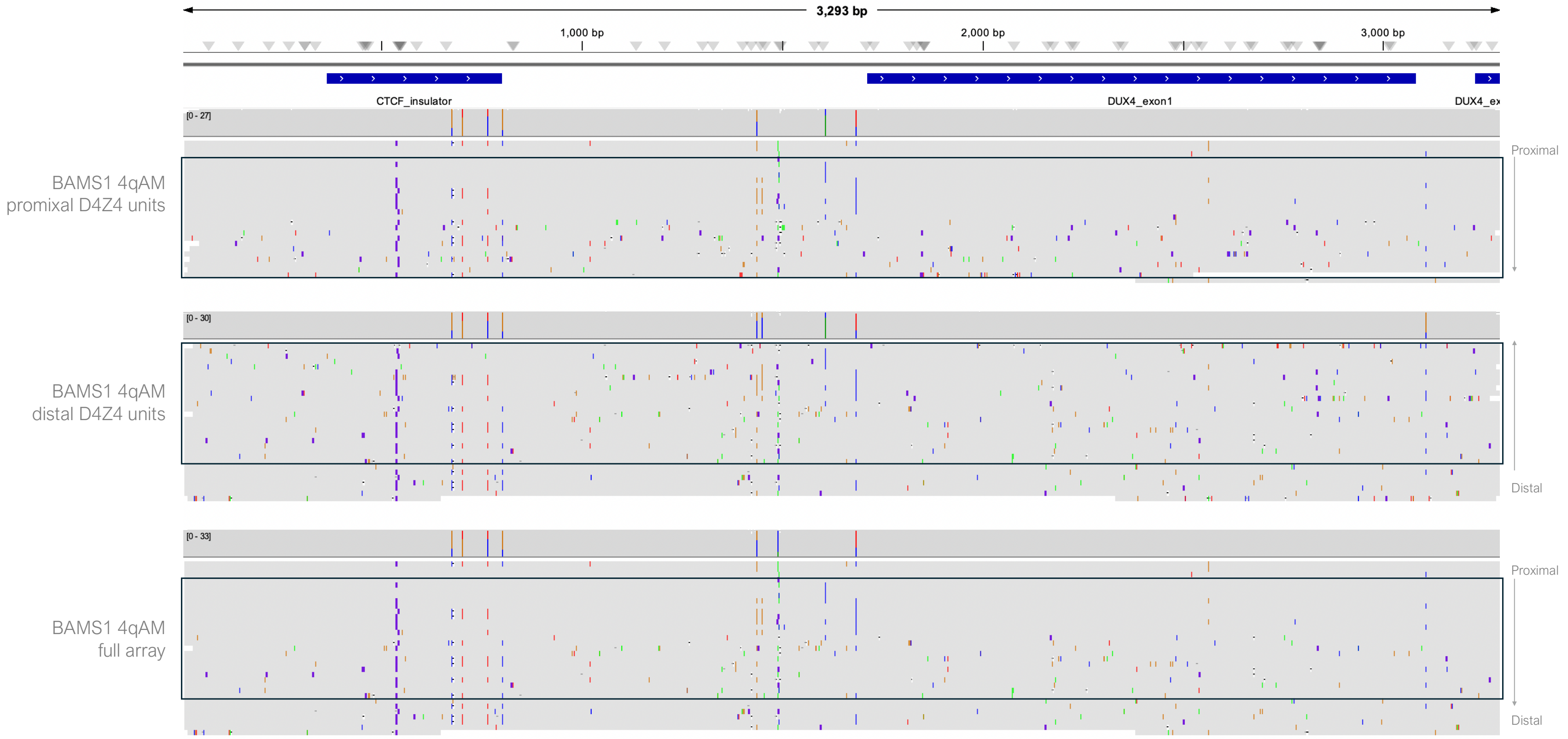

Supplementary Figure S4. Allele-specific analysis of D4Z4 composition enables construction of full-length D4Z4 sequences from non-spanning reads. No spanning 4q reads were obtained for the BAMS1 sample, however variants upstream of D4Z4 enabled 4q reads spanning the proximal end of the array to be phased into two haplotypes using WhatsHap (Martin et al. 2016), while reads spanning the distal end of the array could be phased based on their AS/AM genotype. Consensus sequences were then generated using Racon (Vaser et al. 2017) for each of the groups of phased proximal (top panel) and distal (middle panel) reads. Based on the pattern of variants across the individual D4Z4 units, the region of overlap between the proximal and distal reads was able to be determined (black rectangles) to construct the sequence for the full-length D4Z4 array (bottom panel). The BAMS1 4qAM allele was found to have the same pattern of variants as the 19187_FSHD1 4qAM allele (Supplementary Figure S3C).

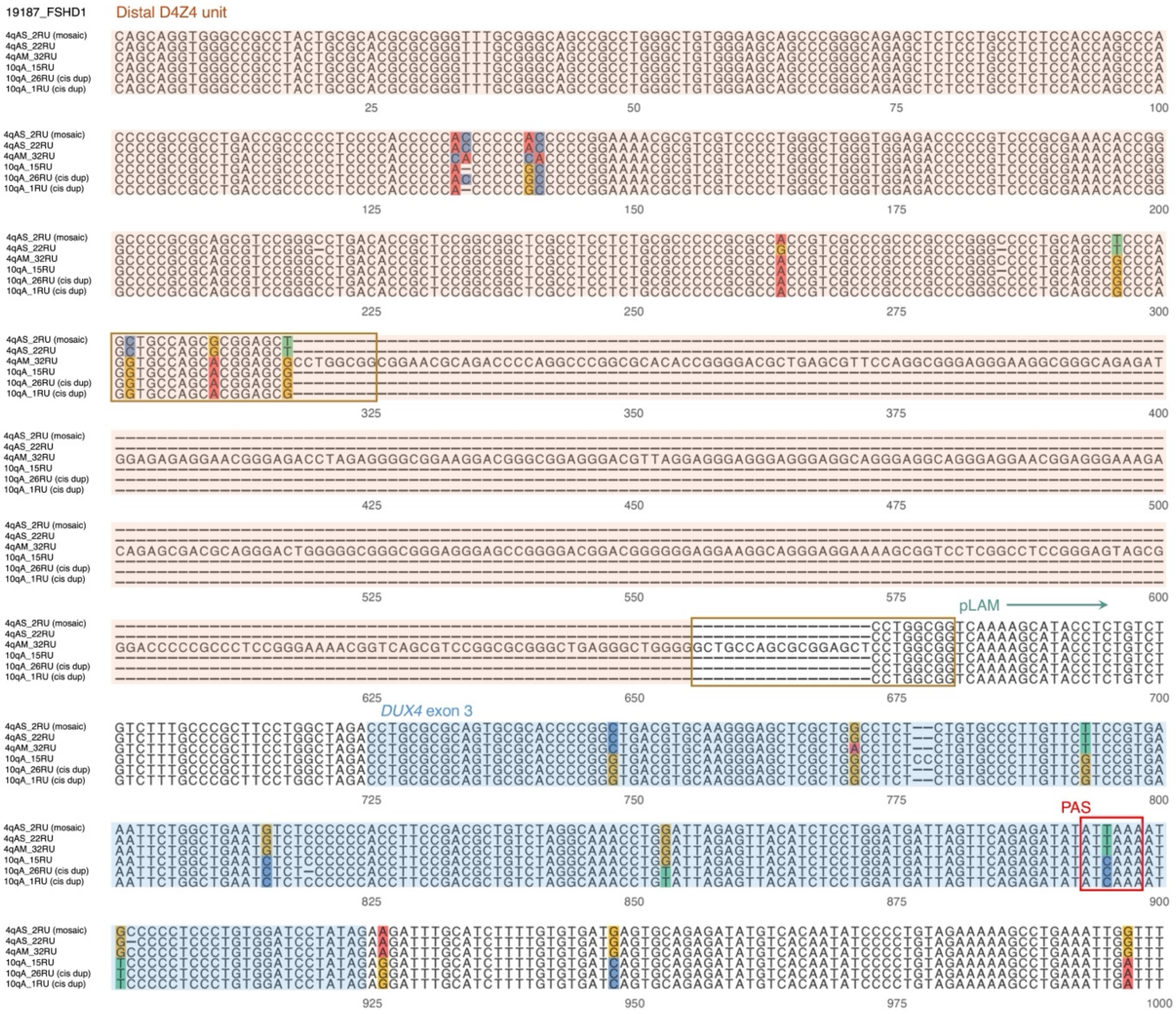

Supplementary Figure S5. Alignment of distal D4Z4 sequences from 4q and 10q alleles from the mosaic FSHD1 sample 19187. 19187 contains 4qAS, 4qAM and 10qA alleles, including an in-*cis* duplicated 10qA array which is also included in the alignment. The orange-boxed areas contain regions of homology between the 4qAM sequence immediately distal to the breakpoint for the final 4qAM D4Z4 unit, and a region within the D4Z4 sequence just proximal to the breakpoint for the 4qAS D4Z4 unit. Here the start of pLAM is marked as the first nucleotide following the final 4qAS D4Z4 unit. Sequences are extracted from consensus sequences generated using Racon (Vaser et al. 2017) from high-coverage Cas9-targeted Nanopore sequencing.

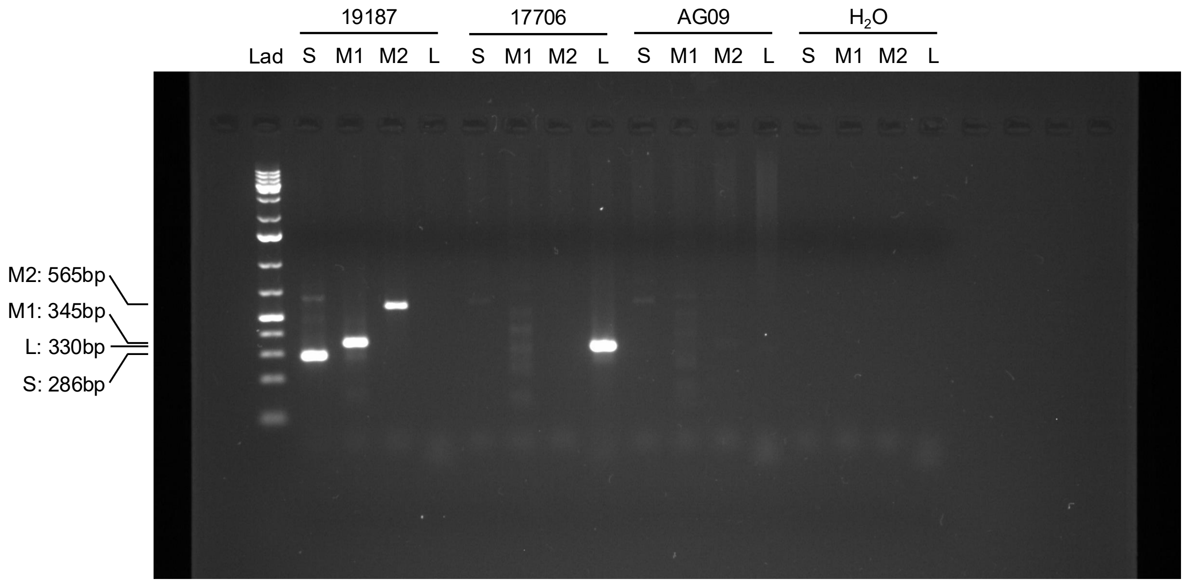

Supplementary Figure S6. PCR genotyping of 4qAS/4qAM/4qAL alleles. PCR reactions used reverse primer PAS-R in conjunction with forward primers specific for 4qAS (S: 4AS-F, 286bp product), 4qAM (M1: 4AM-F_345, 345bp product; M2: 4AM-F_565, 565bp product) or 4qAL (4AL-F, 330bp product) (positions of primers in the D4Z4 distal region shown in Figure 4, sequences in Supplementary Table S5). Sample 19187 (FSHD1, mosaic) has two 4qAS alleles and one 4qAM allele. Sample 17706 (FSHD1, mosaic) has three 4qAL alleles. Sample AG09 (control) has only 4qB alleles. All samples have 10qA alleles, which were not amplified by the primer pairs. Nuclease-free H_2O was used as a negative control. Lad: GeneRuler 1kb Plus DNA ladder.

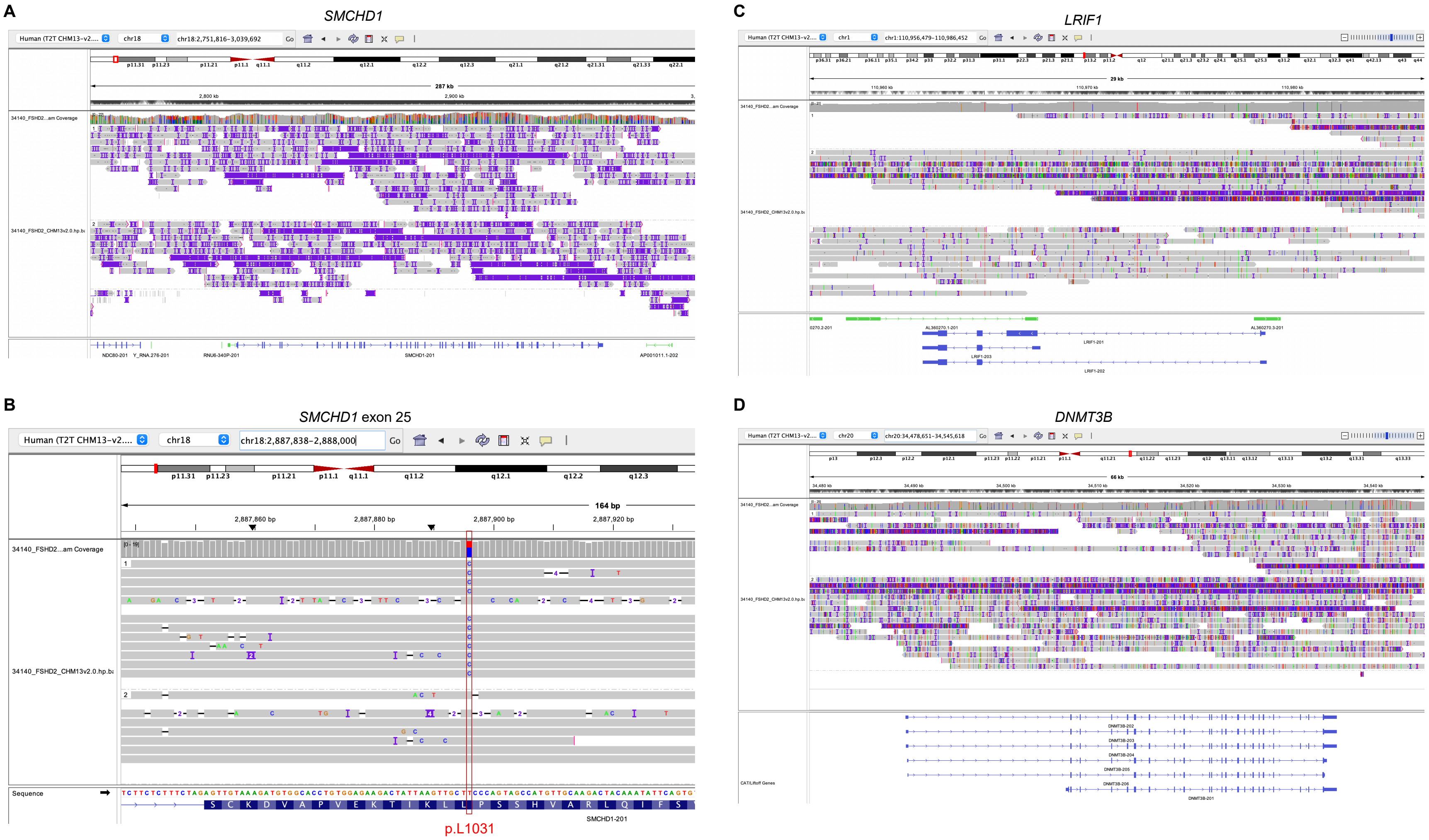

Supplementary Figure S7. IGV screenshots of *SMCHD1, LRIF1* and *DNMT3B* regions for sample 34140. Reads from nanopore ultra-long whole-genome sequencing of sample 34140 were aligned against CHM13v2.0 and phased using WhatsHap (Martin et al. 2016). Sample 34140 has previously been diagnosed as FSHD2, based on clinical findings and the absence of a FSHD1-threshold 4qA allele (1-10 repeat units). Results from long-read sequencing confirm the presence of a previously-identified p.L1031 variant in exon 25 of *SMCHD1* (panel B), however no further single nucleotide variants or structural variants in *SMCHD1, LRIF1* or *DNMT3B* that may contribute to pathogenicity were found. The g.110966369T>C (c.1312A>G) variant in exon 2 of *LRIF1* (panel C) is also found in the other control, FSHD1, FSHD2 and BAMS samples.

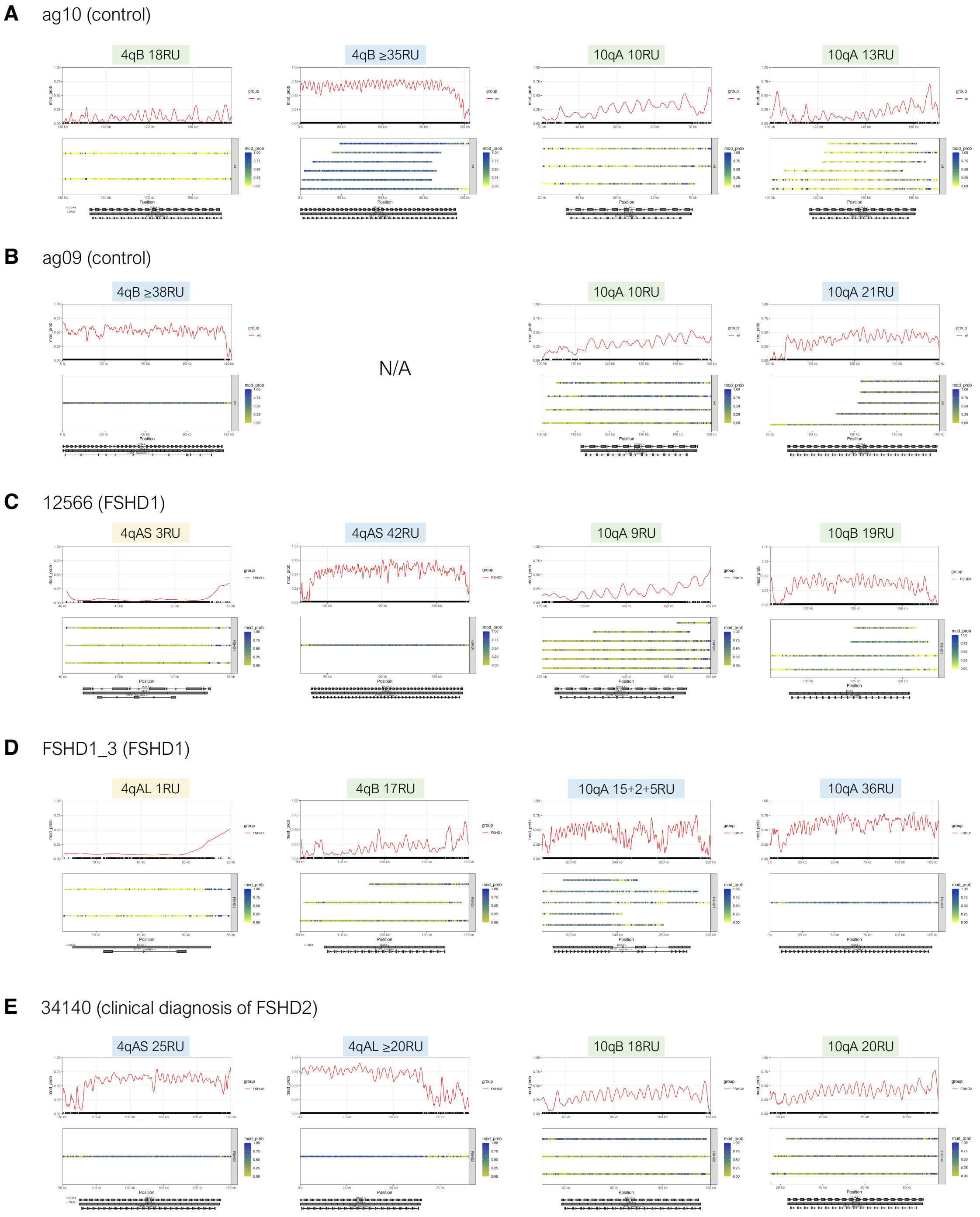

Supplementary Figure S8. Allele-specific, array-wide D4Z4 methylation profiles for *SMCHD1*-wildtype fibroblast samples from whole-genome sequencing data. Single-molecule and smoothed methylation plots for 4q and 10q D4Z4 alleles from (A) ag10: control sample (B) ag09: control sample (C) 12566: FSHD1 sample (D) FSHD1_3: FSHD1 sample (E) 34140: clinically diagnosed as FSHD2, yet lacking pathogenic variants in *SMCHD1*, *LRIF1*, and *DNMT3B*, as determined by Nanopore sequencing. Reads were obtained from ultra-long whole-genome Nanopore sequencing. Methylation plots were generated using NanoMethViz (Su et al. 2021). Annotations for D4Z4 repeat units, CTCF insulator regions, and *DUX4* exons are shown below each plot. Pathogenic 4qA alleles, ‘gray zone’ and intermediate-length alleles (8-20 repeat units), and long alleles (>20 repeat units) are shaded in yellow, green and blue, respectively.

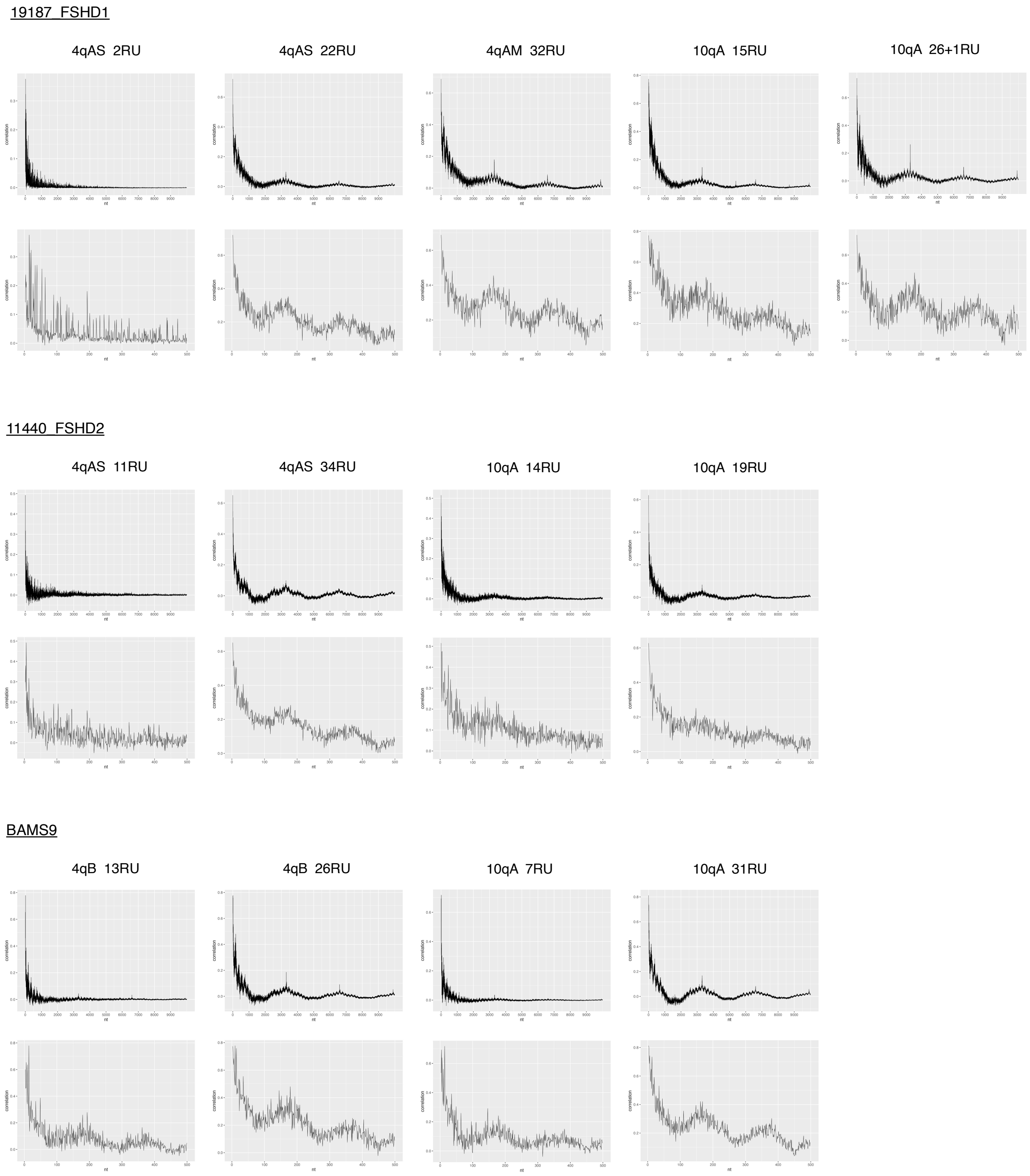

Supplementary Figure S9. Autocorrelation of %5mC for CpG sites across the D4Z4 array for FSHD1, FSHD2 and BAMS alleles. %5mC values are based on the output from modkit v0.2.5. Plots were generated for CpG sites separated by up to 10000nt (top panels) and up to 500nt (bottom panels), and show strong correlation for sites separated by ~3300nt and ~180nt, respectively.

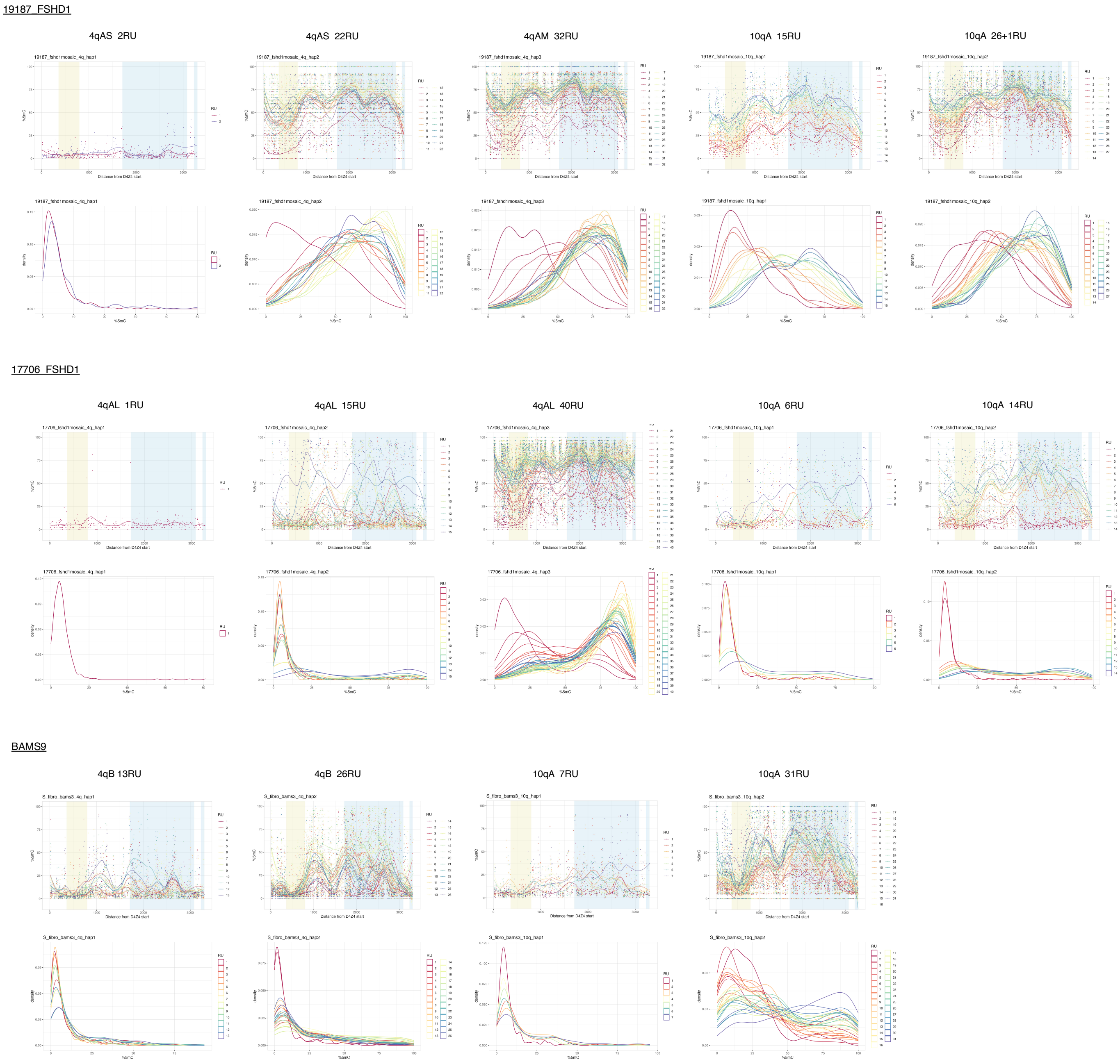

Supplementary Figure S10. %5mC for CpG sites across individual D4Z4 units from FSHD1 and BAMS alleles. The top row for each sample shows plots of the %5mC for each CpG site (individual dots) within each D4Z4 unit from the allele, alongside smoothed lines for each repeat unit. The locations of the CTCF insulator region and *DUX4* exons are shown in yellow and blue, respectively. The bottom row for each sample shows plots of the distributions of %5mC values for each D4Z4 unit from the allele. CpG sites with coverage <5 were filtered out before plotting.

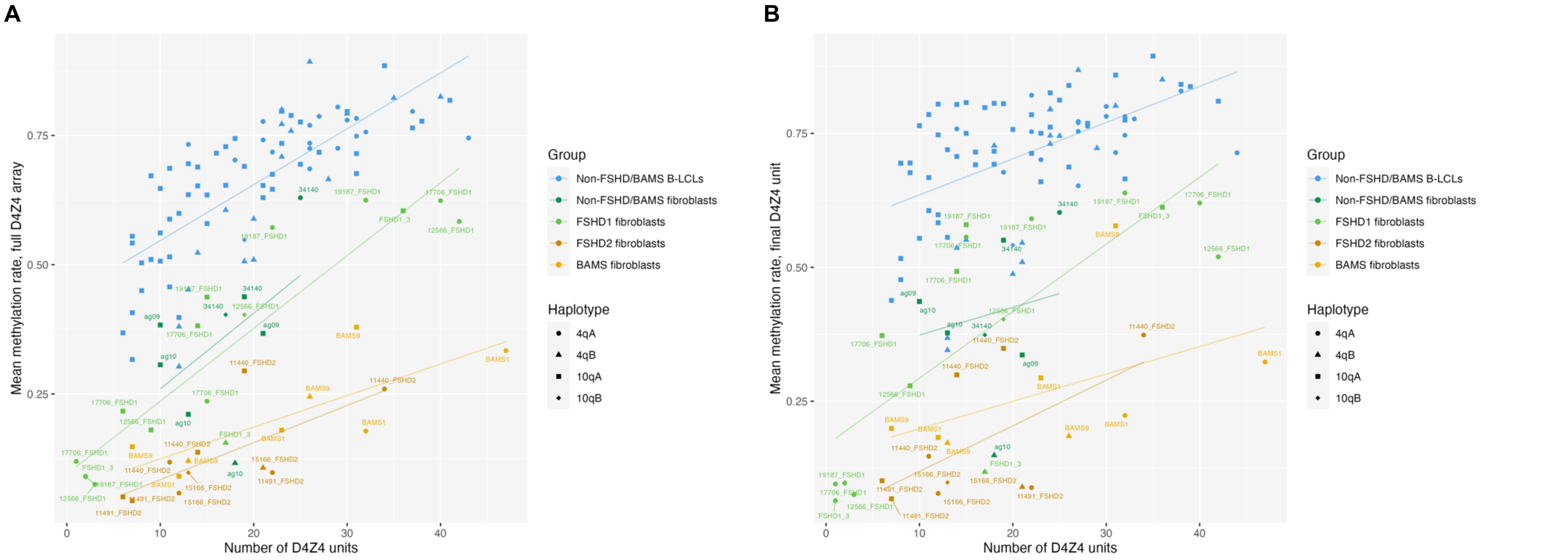

Supplementary Figure S11. **Correlation of overall D4Z4 methylation levels with the number of repeat units, grouped by cell type.** Mean methylation rates across (A) the full 4q/10q D4Z4 array or (B) the final D4Z4 unit against the number of D4Z4 units, plotted for alleles from FSHD1 fibroblasts, non-FSHD/BAMS (control) fibroblasts, FSHD2 fibroblasts, BAMS fibroblasts, and 30 non-FSHD/BAMS B-lymphoblastoid cell lines (B-LCLs) from the 1000 Genomes Project (Gustafson et al. 2024). Mean methylation rate was calculated as the total number of methylated CpGs from all reads / the total number of methylated + unmethylated CpGs from all reads, across either the full D4Z4 array or the final D4Z4 unit. Regression lines were plotted for each group of samples. Sample 34140, while clinically-diagnosed as FSHD2, was grouped with ‘non-FSHD/BAMS fibroblasts’ due to the lack of pathogenic *SMCHD1, DNMT3B* or *LRIF1* variants and lack of FSHD- or BAMS-like hypomethylation.

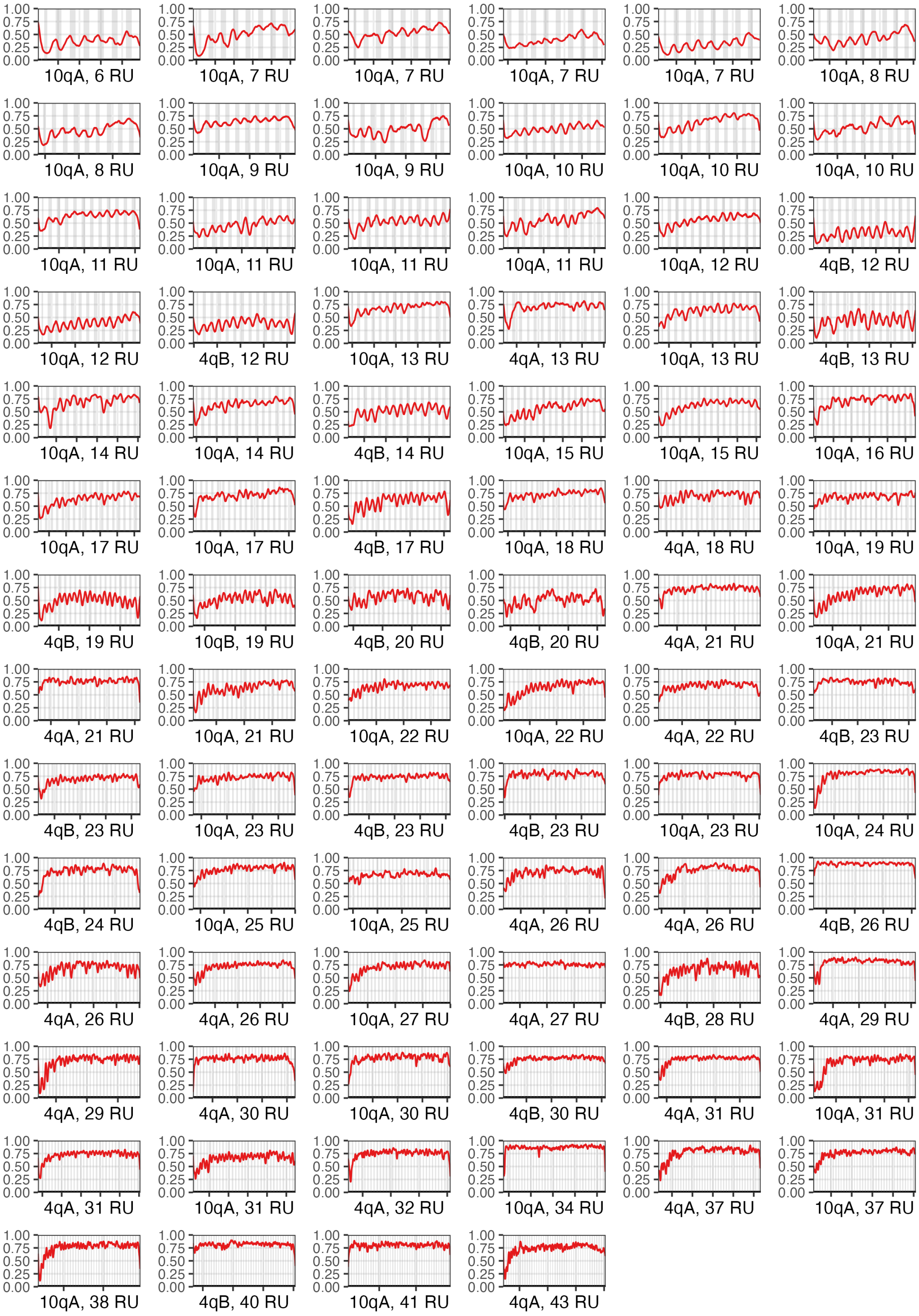

Supplementary Figure S12. Smoothed methylation profiles for 4q and 10q D4Z4 alleles from B-lymphoblastoid cells lines (B-LCLs) from the 1000 Genomes Project. Nanopore sequencing data is from the 1000 Genomes Project ONT Sequencing Consortium (1KGP-ONT) (Gustafson et al. 2024). Smoothed methylation plots were generated from spanning D4Z4 reads using NanoMethViz (Su et al. 2021), in the range of 1.5kb upstream to 1.5kb downstream of the D4Z4 array; the y-axis represents the smoothed methylation probability based on the modification probability stored within the ML tag of the modBAM file. The location of *DUX4* exon 1 within each repeat unit is shown by gray vertical bars.

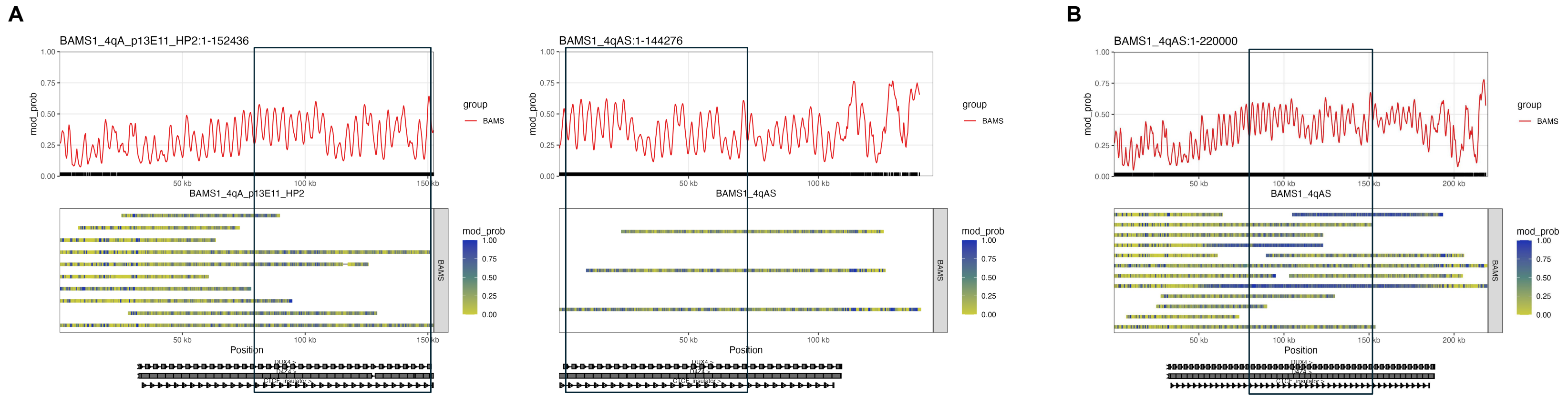

Supplementary Figure S13. Genotype and methylation of the BAMS1 4qAS allele. BAMS1 4qA reads overlapping the proximal end of the array were phased into two groups using WhatsHap (Martin et al. 2016), while 4qA reads overlapping the distal end of the array were phased based on AM/AS haplotype. (A) Smoothed methylation profile and single-molecule methylation plots for proximal D4Z4 reads (left) and distal D4Z4 reads (right) belonging to the BAMS1 4qAS allele, excluding hypermethylated reads. The presumed area of overlap between the proximal and distal reads (black rectangle) was determined based on the smoothed methylation profile. (B) Smoothed methylation profile and single-molecule methylation plots for proximal and distal D4Z4 4qAS reads aligned to a reconstructed full-length D4Z4 reference sequence with 47 repeat units. Four reads that mapped to the 4qAM haplotype had very high methylation levels compared to the other reads. The area of overlap between the proximal and distal reads is shown in the black rectangle.

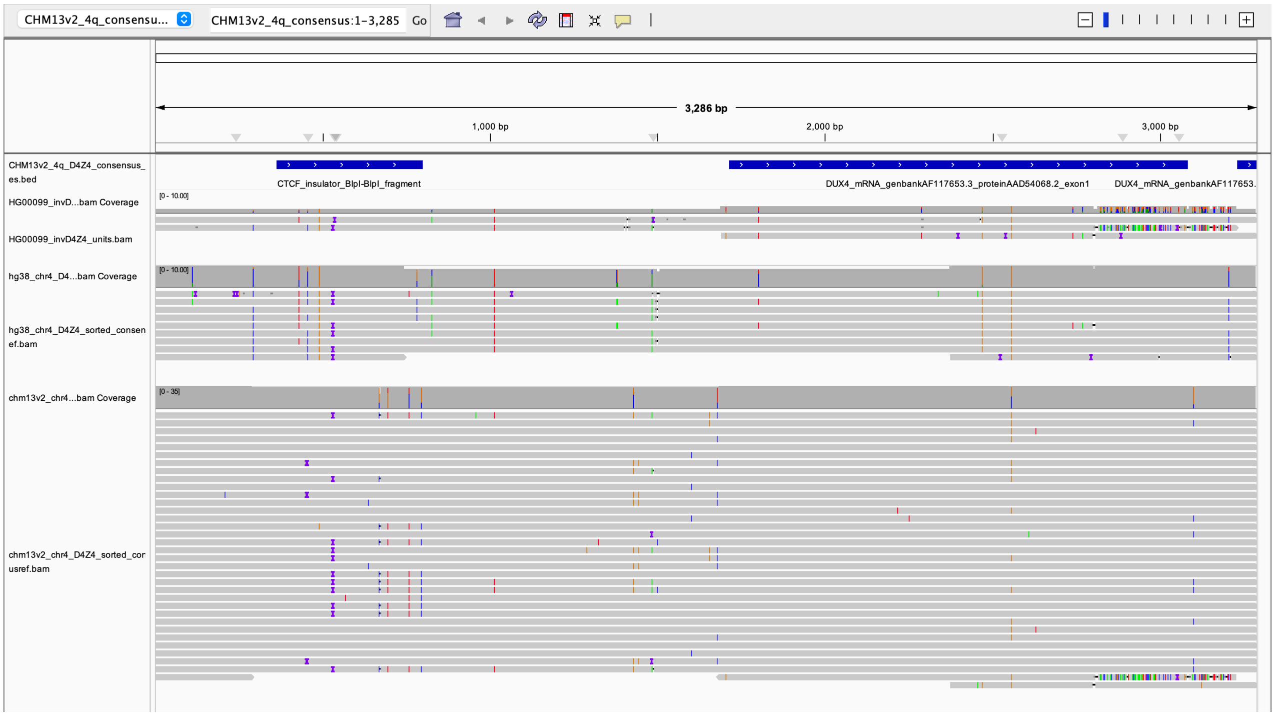

Supplementary Figure S14. D4Z4 composition of the upstream inverted array on the HG00099 4qB allele. Sequences of D4Z4 units were extracted from consensus sequences generated for the upstream inverted array using Racon (Vaser et al. 2017), and aligned against a 4qA-type D4Z4 reference sequence (top track). Alignments of D4Z4 units from the GRCh38 4qB array (middle track) and the CHM13v2.0 4qA array (bottom track) are also shown, showing that the first (partial) and second (full) D4Z4 units of the upstream inverted array are 4qB-type, while the second half of the third (full) D4Z4 unit of the upstream inverted array corresponds to the normal sequence for the D4S2463 unit.

References

Butterfield RJ, Dunn DM, Duval B, Moldt S, Weiss RB. 2023. Deciphering D4Z4 CpG methylation gradients in fascioscapulohumeral muscular dystrophy using nanopore sequencing. *Genome Res* **33**: 1439–1454.

Delourme M, Charlene C, Gerard L, Ganne B, Perrin P, Vovan C, Bertaux K, Nguyen K, Bernard R, Magdinier F. 2023. Complex 4q35 and 10q26 Rearrangements: A Challenge for Molecular Diagnosis of Patients With Facioscapulohumeral Dystrophy. *Neurol Genet* **9**: e200076.

Dion C, Roche S, Laberthonnière C, Broucqsault N, Mariot V, Xue S, Gurzau AD, Nowak A, Gordon CT, Gaillard M-C, et al. 2019. SMCHD1 is involved in *de novo* methylation of the *DUX4* -encoding D4Z4 macrosatellite. *Nucleic Acids Research* **47**: 2822–2839.

Dixit M, Ansseau E, Tassin A, Winokur S, Shi R, Qian H, Sauvage S, Mattéotti C, van Acker AM, Leo O, et al. 2007. DUX4, a candidate gene of facioscapulohumeral muscular dystrophy, encodes a transcriptional activator of PITX1. *Proceedings of the National Academy of Sciences* **104**: 18157–18162.

Gaillard M-C, Broucqsault N, Morere J, Laberthonnière C, Dion C, Badja C, Roche S, Nguyen K, Magdinier F, Robin JD. 2019. Analysis of the 4q35 chromatin organization reveals distinct long-range interactions in patients affected with Facio-Scapulo-Humeral Dystrophy. *Sci Rep* **9**: 10327.

Gérard L, Delourme M, Tardy C, Ganne B, Perrin P, Chaix C, Trani JP, Eudes N, Laberthonnière C, Bertaux K, et al. 2024. SMCHD1 genetic variants in type 2 facioscapulohumeral dystrophy and challenges in predicting pathogenicity and disease penetrance. *Eur J Hum Genet* **33**: 784–792

Gordon CT, Xue S, Yigit G, Filali H, Chen K, Rosin N, Yoshiura K, Oufadem M, Beck TJ, McGowan R, et al. 2017. De novo mutations in SMCHD1 cause Bosma arhinia microphthalmia syndrome and abrogate nasal development. *Nat Genet* **49**: 249–255.

Gustafson JA, Gibson SB, Damaraju N, Zalusky MPG, Hoekzema K, Twesigomwe D, Yang L, Snead AA, Richmond PA, Coster WD, et al. 2024. High-coverage nanopore sequencing of samples from the 1000 Genomes Project to build a comprehensive catalog of human genetic variation. *Genome Res* **34**: 2061–2073.

Hiramuki Y, Kure Y, Saito Y, Ogawa M, Ishikawa K, Mori-Yoshimura M, Oya Y, Takahashi Y, Kim D-S, Arai N, et al. 2022. Simultaneous measurement of the size and methylation of chromosome 4qA-D4Z4 repeats in facioscapulohumeral muscular dystrophy by long-read sequencing. *J Transl Med* **20**: 517.

Huang M, Zhang Q, Jiao J, Shi J, Xu Y, Zhang C, Zhou R, Liu W, Liang Y, Chen H, et al. 2024. Comprehensive genetic analysis of facioscapulohumeral muscular dystrophy by Nanopore long-read whole-genome sequencing. *J Transl Med* **22**: 451.

Labun K, Montague TG, Krause M, Torres Cleuren YN, Tjeldnes H, Valen E. 2019. CHOPCHOP v3: expanding the CRISPR web toolbox beyond genome editing. *Nucleic Acids Research* **47**: W171–W174.

Lemmers RJ, van der Vliet PJ, Balog J, Goeman JJ, Arindrarto W, Krom YD, Straasheijm KR, Debipersad RD, Özel G, Sowden J, et al. 2018. Deep characterization of a common D4Z4 variant identifies biallelic DUX4 expression as a modifier for disease penetrance in FSHD2. *Eur J Hum Genet* **26**: 94–106.

Lemmers RJLF, Butterfield R, Van Der Vliet PJ, De Bleecker JL, Van Der Pol L, Dunn DM, Erasmus CE, D'Hooghe M, Verhoeven K, Balog J, et al. 2024. Autosomal dominant *in cis* D4Z4 repeat array duplication alleles in facioscapulohumeral dystrophy. *Brain* **147**: 414–426.

Li K, Quiat D, She F, Liu Y, He R, Haghighi A, Liu F, Zhang R, DePalma SR, Yang Y, et al. 2024. Genetic diagnosis of facioscapulohumeral muscular dystrophy type 1 using rare-variant linkage analysis and long-read genome sequencing. *Genetics in Medicine Open* **2**: 101817. 10.1016/j.gimo.2024.101817.

Liao WW, Asri M, Ebler J, Doerr D, Haukness M, Hickey G, Lu S, Lucas JK, Monlong J, Abel HJ, et al. 2023. A draft human pangenome reference. *Nature* **617**: 312–324.

Martin M, Patterson M, Garg S, O Fischer S, Pisanti N, Klau GW, Schöenhuth A, and Marschall T. 2016. Whatshap: fast and accurate read-based phasing. bioRxiv doi: 10.1101/085050.

Su S, Gouil Q, Blewitt ME, Cook D, Hickey PF, Ritchie ME. 2021. NanoMethViz: An R/Bioconductor package for visualizing long-read methylation data. *PLOS Computational Biology* **17**: e1009524. 10.1371/journal.pcbi.1009524

Vaser R, Sović I, Nagarajan N, Šikić M. 2017. Fast and accurate de novo genome assembly from long uncorrected reads. *Genome Res* **27**: 737–746.

Wang Y, Zhao Z, Meng F, Kong X. 2024. Accurate prenatal diagnosis of facioscapulohumeral muscular dystrophy 1 using nanopore sequencing. *J Med Genet* **61**: 1096–1102.

Ye J, Coulouris G, Zaretskaya I, Cutcutache I, Rozen S, Madden TL. 2012. Primer-BLAST: a tool to design target-specific primers for polymerase chain reaction. *BMC Bioinformatics* **13**: 134.

Yeetong P, Kulsirichawaroj P, Kumutpongpanich T, Srichomthong C, Od-ek P, Rakwongkhachon S, Thamcharoenvipas T, Sanmaneechai O, Pongpanich M, Shotelersuk V. 2023. Long-read Nanopore sequencing identified D4Z4 contractions in patients with facioscapulohumeral muscular dystrophy. *Neuromuscular Disorders* **33**: 551–556.
