# Supplementary figures and images for: Complete genetic and epigenetic architecture of D4Z4 macrosatellites in FSHD, BAMS, and reference cohorts with D4Z4End2End
Source: Genome Res. 2026 Apr;36(4):827–48. doi: 10.1101/gr.280907.125 (PMC13138017; doi:10.1101/gr.280907.125)

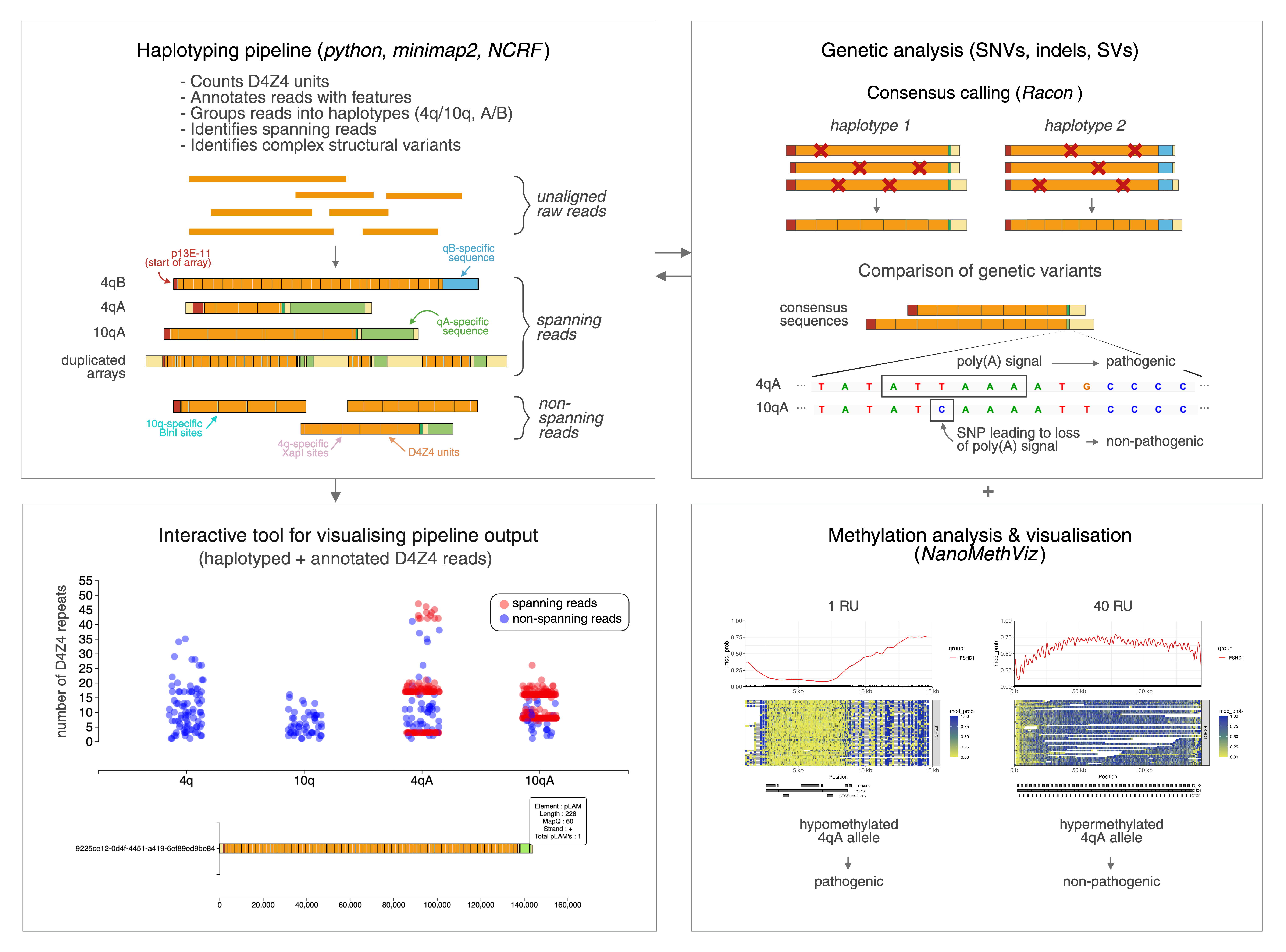

Supplement: Supplement 2 [file Supplemental_code.zip › Supplemental_code/figures/D4Z4End2End.png]

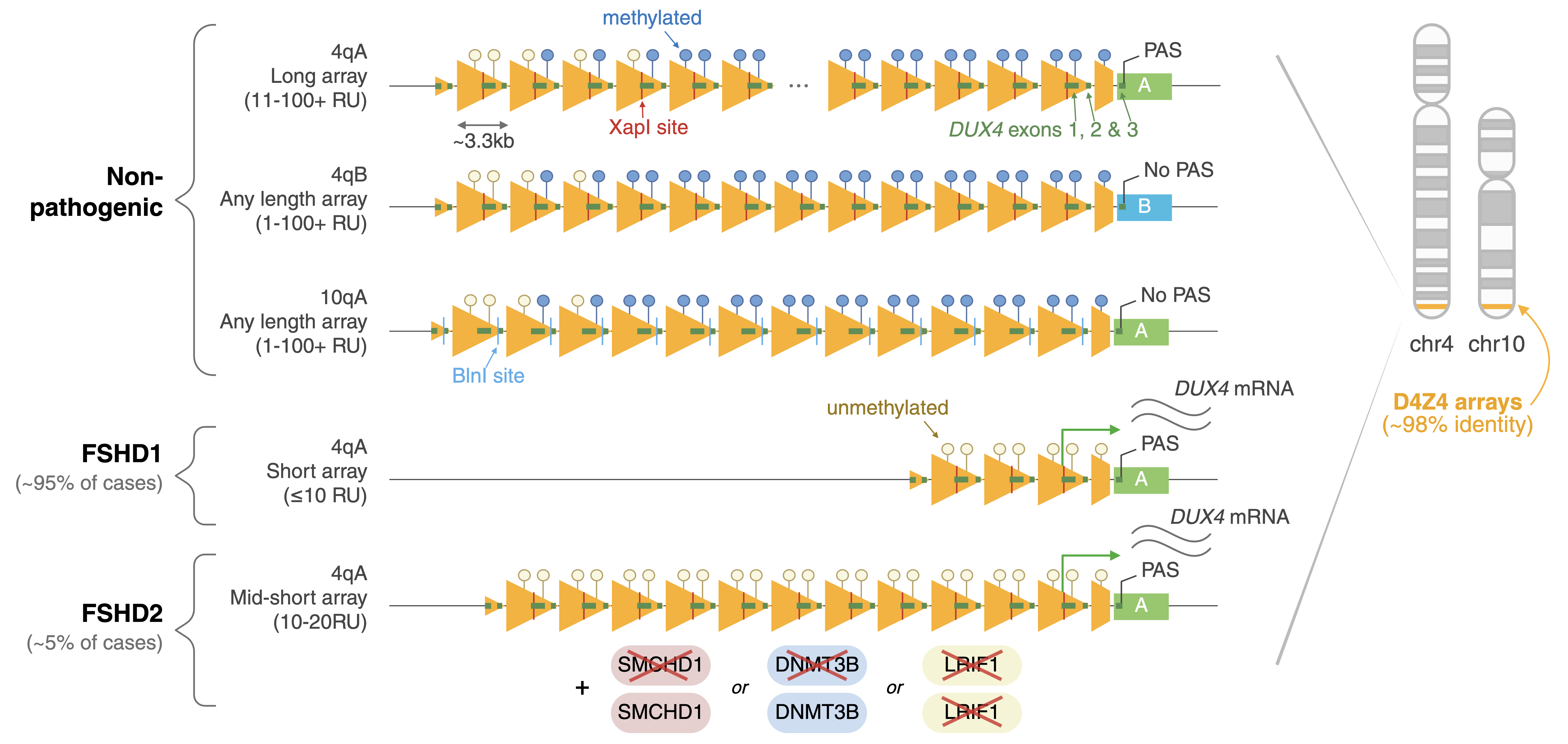

Supplement: Supplement 2 [file Supplemental_code.zip › Supplemental_code/figures/FSHD_pathogenesis.png]

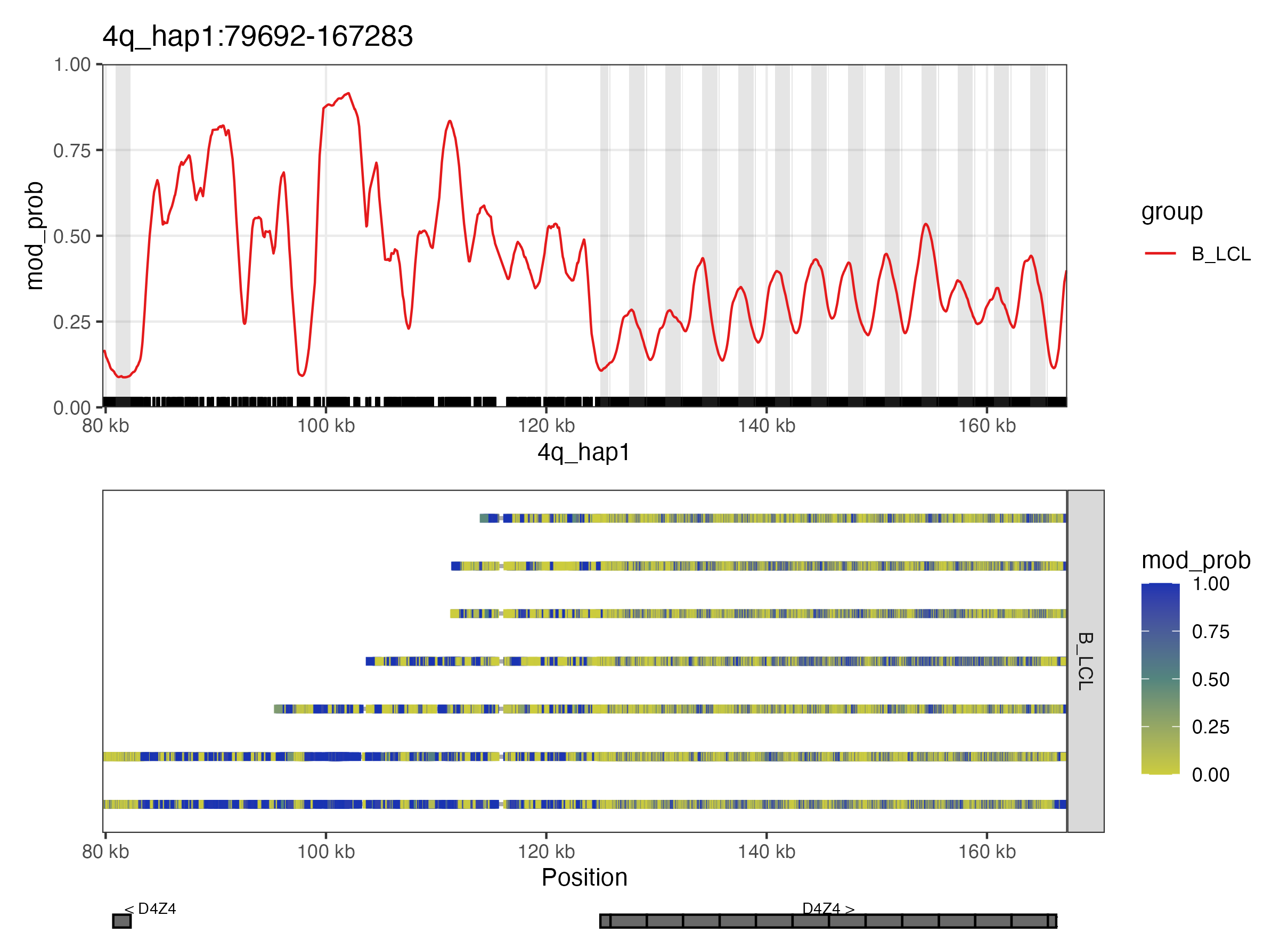

Supplement: Supplement 2 [file Supplemental_code.zip › Supplemental_code/figures/HG02185_4q_hap1.png]

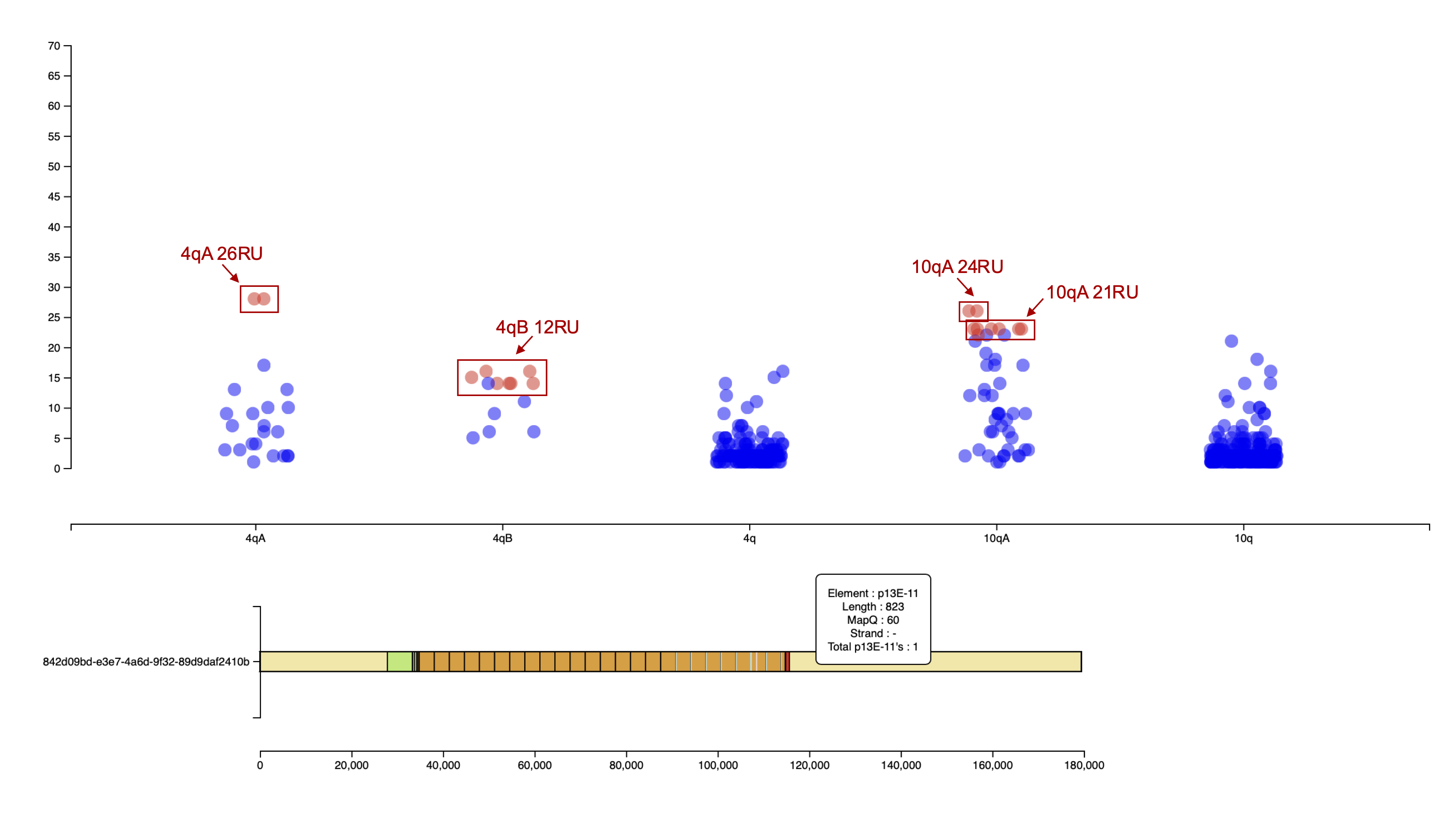

Supplement: Supplement 2 [file Supplemental_code.zip › Supplemental_code/figures/HG02185_haplo_raw.png]

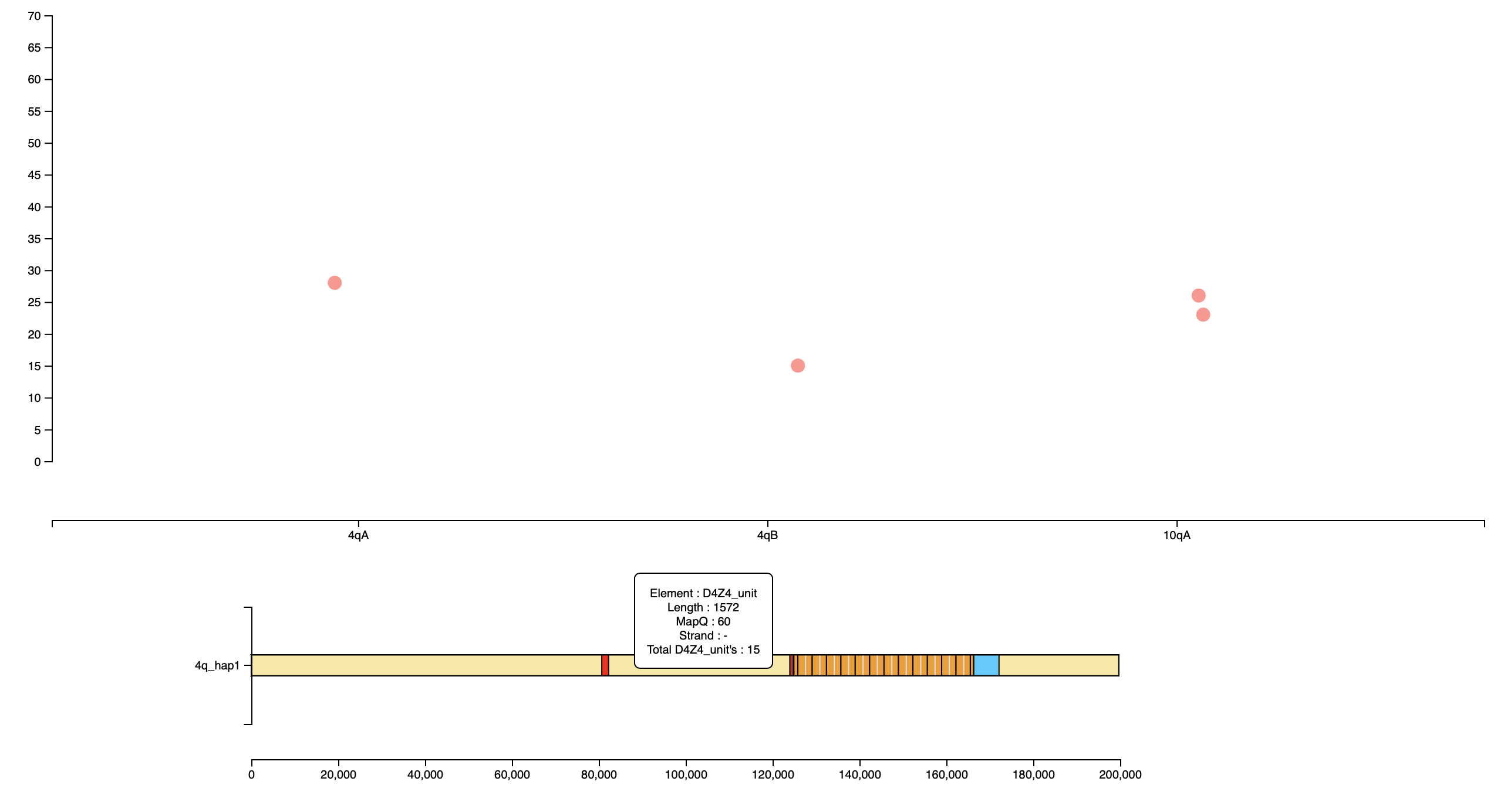

Supplement: Supplement 2 [file Supplemental_code.zip › Supplemental_code/figures/HG02185_haplo_ref.png]
